# Association Between Dietary Index for Gut Microbiota and the Risk of Pelvic Inflammatory Disease: Mediating Role of Anti‐ and Pro‐Inflammatory Dietary Patterns

**DOI:** 10.1002/fsn3.71563

**Published:** 2026-02-19

**Authors:** Yanjing Bao, Xianyue Hu, Tianyang Gao, Wenfeng Hua

**Affiliations:** ^1^ The Second School of Clinical Medicine Southern Medical University Guangzhou People's Republic of China; ^2^ Department of Reproductive Medicine Center, the Affiliated Guangdong Second Provincial General Hospital of Jinan University Guangzhou People's Republic of China; ^3^ Department of Gynecology, the Affiliated Guangdong Second Provincial General Hospital of Jinan University Guangzhou People's Republic of China; ^4^ Southern Medical University Guangzhou People's Republic of China; ^5^ Research Institute for Maternal and Child Health, the Affiliated Guangdong Second Provincial General Hospital of Jinan University Guangzhou People's Republic of China

**Keywords:** dietary index for gut microbiota, dietary inflammation index, gut microbiota, mediation, NHANES, pelvic inflammatory disease

## Abstract

Recent studies suggest that an imbalance in the gut microbiota, known as dysbiosis, may affect inflammation. The Dietary Index for Gut Microbiota (DI‐GM) is a new measure of dietary quality that reflects gut microbiota diversity and abundance. However, its association with pelvic inflammatory disease (PID) remains unclear. This study explored the relationship between DI‐GM and the risk of developing PID. This cross‐sectional study analyzed data from 4539 women aged 18–59 years obtained from the National Health and Nutrition Examination Survey (NHANES) database for the period of 2013–2018. To explore the association between DI‐GM and PID, we employed weighted multivariable linear and logistic regression, restricted cubic splines (RCS), and subgroup analyses. Additionally, mediation analysis was conducted to assess the influence of anti‐ and pro‐inflammatory dietary patterns, represented by the Dietary Inflammatory Index (DII), on the relationship between DI‐GM and PID. Among the eligible participants, 255 (5.62%) had PID. A higher proportion of participants with lower DI‐GM scores experienced PID. Multivariable logistic regression analysis revealed a negative association between DI‐GM and the risk of PID, regardless of whether the independent variable was considered a continuous variable or in quartiles in the fully adjusted model (Model 3, continuous variable: OR = 0.87, 95% confidence interval (CI): 0.79–0.96, *p* = 0.012; Q3 vs. Q1: OR = 0.58, 95% CI = 0.36–0.94, *p* = 0.036; Q4 vs. Q1: OR = 0.55, 95% CI = 0.35–0.87, *p* = 0.017, *p* for trend = 0.018). The RCS curves demonstrated a non‐linear relationship between the DI‐GM scores and PID risk. Subgroup analyses indicated an inverse correlation between DI‐GM and PID risk across various covariates. Mediation analysis showed that inflammatory dietary patterns accounted for 26.82% of the mediation proportion in the association between DI‐GM and PID. These results indicate that higher DI‐GM scores are correlated with a decreased risk of PID. Inflammatory dietary patterns may mediate the association between DI‐GM and PID, suggesting that restoring gut homeostasis and health through dietary interventions may prevent or ameliorate PID.

## Graphical abstract

Higher DI‐GM scores were associated with a lower risk of developing PID. Inflammatory dietary patterns may influence the relationship between DI‐GM and PID, indicating that improving gut health through dietary changes may prevent or alleviate PID.

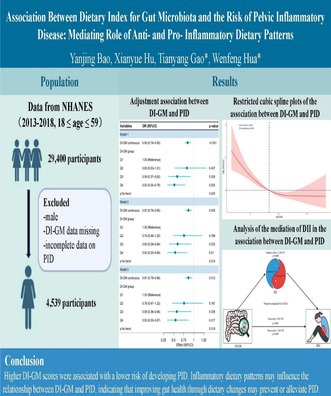

## 1 Introduction

Pelvic inflammatory disease (PID) is a prevalent infectious condition that affects the upper reproductive system in females and is generally categorized as acute or chronic (Brunham et al. 2015; Ford and Decker 2016). This disease affects the uterus, fallopian tubes, and/or ovaries, potentially resulting in long‐term reproductive issues or severe complications such as infertility, ectopic pregnancy, and chronic pelvic pain. Moreover, many women with PID exhibit subtle symptoms and signs, and some may experience a clinically silent infection that spreads to the upper genital tract, leading to subclinical PID (Brunham et al. 2015; Ford and Decker 2016). Recently, there has been a shift toward an earlier onset, which has a considerable impact on women's reproductive health and overall quality of life, endangering both their physical and mental well‐being (Kreisel et al. 2017).

The primary treatment for acute PID involves intravenous, intramuscular, or oral administration of broad‐spectrum antibiotics. However, the prolonged and extensive use of these antibiotics can affect physical health and contribute to increased bacterial resistance (Ravel et al. 2021; Savaris et al. 2020). Recent studies have demonstrated that the gut microbiota plays a crucial role in maintaining host health. It contributes to the provision of various nutrients, maintenance of metabolic balance, regulation of the host immune system, and reduction of systemic inflammation (Pei et al. 2025; Sharma et al. 2025; Yoo et al. 2020). Several studies have found that the gut microbiota may play a significant role in modulating PID development (Dong et al. 2025; Ji et al. 2024; Yin et al. 2025). Maintaining a favorable balance of gut microbiota is advantageous for host health. Studies have indicated that dietary habits influence the gut microbiota composition, affecting intestinal microbiota homeostasis, gut barrier integrity, and immune response (Perrone and D'Angelo 2025). These factors collectively contribute to host health by preventing pathogen invasion and reducing the risk of recurrent infections (Bailey and Holscher 2018; Gibiino et al. 2021; Zhao and Zhao 2023). The link between gut microbiota and dietary habits indicates that dietary modifications and nutritional supplements could be promising intervention strategies for treating PID (Perrone and D'Angelo 2025).

The dietary index for gut microbiota (DI‐GM) was created to reflect the diversity and abundance of gut microbiota according to dietary quality. This index has been validated using markers indicative of the gut microbiota. Unlike traditional dietary evaluation metrics, the DI‐GM systematically evaluates the regulatory impacts of 14 food and nutrient categories (encompassing 10 beneficial components and four detrimental components), thereby facilitating a quantitative assessment of the gut microbiota health status (Kase et al. 2024). Recent studies have shown that DI‐GM provides a novel quantitative method for exploring the relationships between diet, gut microbiota, and disease (S. Hu et al. 2025; Meng et al. 2025; Pu et al. 2025; Shu et al. 2025; Zheng et al. 2025). However, the relationship between the DI‐GM and PID remains ambiguous. Notably, research has demonstrated that the Dietary Inflammatory Index (DII), a rigorously validated instrument for assessing dietary inflammation by evaluating nutrient consumption based on anti‐inflammatory or pro‐inflammatory properties, is associated with various diseases, including PID (Ma et al. 2024; Ren et al. 2025; Tang et al. 2024; X. Wang et al. 2025). Moreover, inflammation and oxidative stress have a reciprocal relationship, in which inflammation leads to an increase in reactive oxygen species (ROS) production, which in turn intensifies inflammation (Alijani et al. 2024; Zheng et al. 2024). These findings suggest that anti‐ and pro‐inflammatory dietary patterns may influence chronic inflammatory diseases by modulating gut microbiota. This cross‐sectional study aimed to fill this knowledge gap by investigating the association between DI‐GM, DII, and PID using the National Health and Nutrition Examination Survey (NHANES) data. Additionally, this study sought to offer valuable insights into the development of targeted dietary interventions to mitigate PID.

## 2 Methods

### 2.1 Data Source

This study employed data from the 2013–2018 NHANES, a comprehensive cross‐sectional survey conducted biennially to collect demographic, socioeconomic, dietary, and health information from the non‐institutionalized population of the United States using multistage probability sampling. The data are publicly accessible through the National Center for Health Statistics (NCHS), a division of the Centers for Disease Control and Prevention (CDC). All NHANES survey protocols were approved by the Institutional Review Board of the NCHS, and all participants provided written informed consent before survey initiation.

### 2.2 Study Design and Population

**FIGURE 1:**
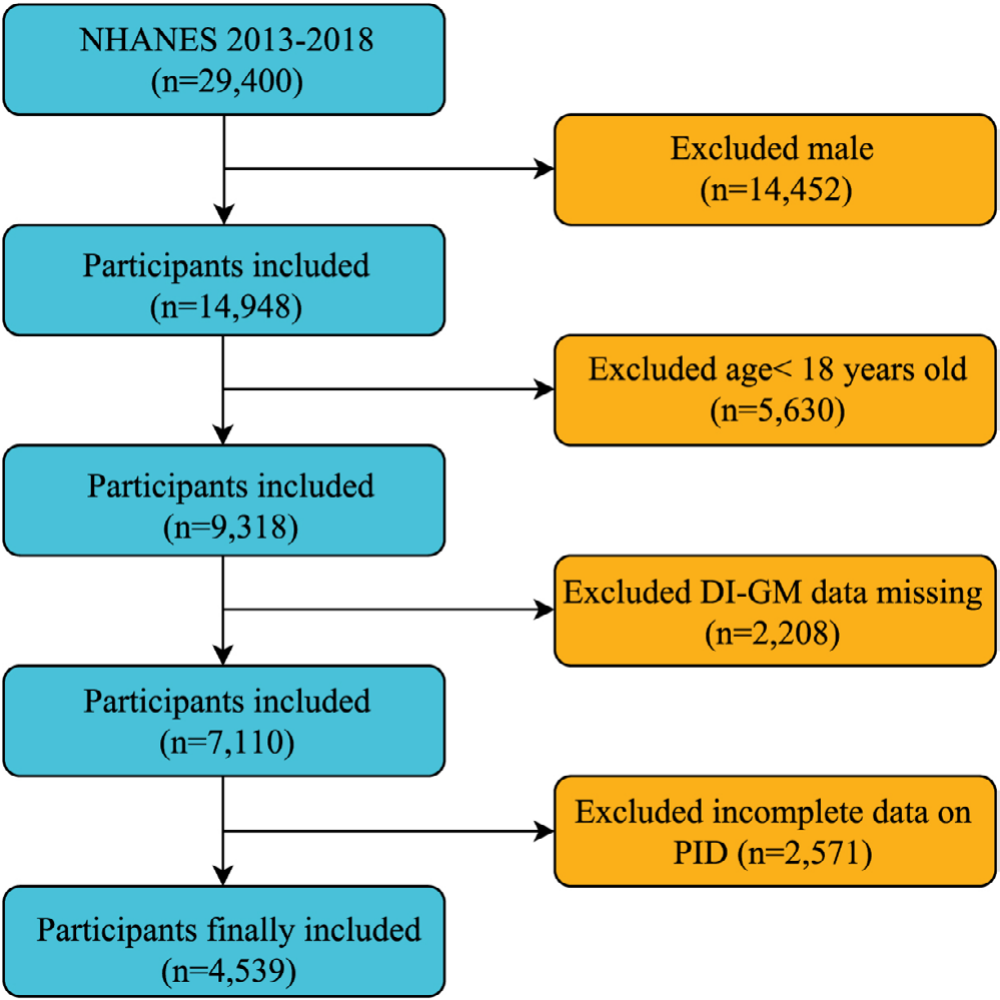
Flow chart of the inclusion and exclusion of study participants.

The study sample consisted of women aged 18–59 years. A comprehensive dataset, including DI‐GM components and PID status, was obtained from an initial cohort of 29,400 participants. Several groups were excluded from the study: males (*n* = 14,452), those aged < 18 years (*n* = 5630), individuals lacking DI‐GM components (*n* = 2208), and those without PID information (*n* = 2571). Consequently, the final analysis included 4539 participants (Figure 1).

### 2.3 Calculation of DI‐GM

The dietary index for DI‐GM is an innovative tool for evaluating dietary quality by examining the effects of dietary components on gut microbiota diversity. This index encompasses 14 foods and nutrients theoretically linked to intestinal flora health, including ten beneficial items (avocado, broccoli, chickpeas, coffee, cranberries, fermented dairy products, fiber, green tea, soy, and whole grains) and four detrimental items (red meat, processed meat, refined grains, and a high‐fat diet, defined as ≥ 40% of total energy from fat). Intake data were gathered from the average of two 24 h dietary recall interviews, excluding participants with only one reliable dietary recall. A score of 1 was assigned for consumption above the median for beneficial components and below the median for harmful components, whereas a score of 0 indicated the opposite (Kase et al. 2024). The total score ranged from 0 to 13, with higher scores indicating healthier gut microbiota. Based on our previous research (Zhang, Wu, et al. 2025), the participants were categorized into four groups: 0–3, 4, 5, and ≥ 6.

### 2.4 Definition of PID

PID diagnosis was evaluated using data from the NHANES Reproductive Health Questionnaire. Specifically, question RHQ078 inquired: “Have you ever been treated for an infection in your fallopian tubes, uterus, or ovaries, also called a pelvic infection, pelvic inflammatory disease, or PID?” Respondents who answered affirmatively were classified as having PID.

### 2.5 Calculation of DII

DII was systematically calculated using a pre‐validated formula, with the objective of comprehensively quantifying the inflammatory potential of dietary patterns across diverse populations (Shivappa et al. 2014). For each individual, the intake of specific food components was initially compared with the global mean intake of the respective components. Subsequently, z‐scores were derived by normalizing the difference between an individual's self‐reported intake and the global mean, using the corresponding standard deviation. Consistent with the methodologies described in previous studies, these z‐scores were further transformed into standardized percentiles and adjusted to account for the differential inflammatory contributions of each food‐related parameter (Zheng et al. 2024). Finally, the DII values corresponding to all food components were aggregated to generate a composite DII score, where elevated values indicated a dietary pattern with increased proinflammatory characteristics.

### 2.6 Covariates

The covariables of interest encompassed sociodemographic, lifestyle, and health information, identified a priori as potential confounding factors based on previous studies (Chen et al. 2024; Hu, Zhang, et al. 2023; Hu, Hu, et al. 2023; Zhang et al. 2023; D. Wang and Xiong 2025). These included demographic characteristics such as age, race, marital status, education level, and poverty income ratio; lifestyle behaviors such as alcohol consumption and smoking; health conditions, including body mass index (BMI), hypertension, and diabetes; and reproductive health factors, notably the regularity of menstrual cycles. Detailed information on the methodologies used for data collection of these variables can be found on the official NHANES website (https://www.cdc.gov/nchs/nhanes/index.html).

### 2.7 Statistical Analysis

Continuous variables are presented as means with standard errors (SE), and categorical variables are expressed as numerical counts and percentage frequencies (%). To evaluate the differences between the PID and non‐PID groups, a weighted chi‐square test was applied to categorical variables, ANOVA to normally distributed variables, and the Kruskal‐Wallis H test to skewed distributions. For variables with missing data, multivariate multiple imputations (MI) were utilized, employing five replications and chained equations within the R MI procedure to address missing data effectively. The proportion of missing data varied across different variables: BMI (0.82%), education (0.02%), marital status (7.60%), PIR (8.24%), smoking (0.02%), drinking (19.43%), diabetes (0.09%), and hypertension (0.04%). Three statistical models were developed for each of the analyses. The crude model (Model 1) was not adjusted. Model 2 was adjusted for age, ethnicity, education level, PIR, and marital status. Model 3 included further adjustments for BMI, smoking status, alcohol consumption, diabetes mellitus, hypertension, and regular menstrual cycle. Weighted generalized linear and multivariate logistic regression models were used to examine the associations between DI‐GM, DII, and PID. Subgroup analyses were conducted to explore the association between DI‐GM and PID across various demographic and clinical groups, considering factors such as age, BMI, PIR, alcohol consumption, smoking, menstrual regularity, hypertension, and diabetes mellitus.

Additionally, restricted cubic spline (RCS) curves and threshold effect analyses were employed to investigate the potential nonlinear relationship between DI‐GM, DII, and PID risk. Finally, mediation analysis was performed to determine whether the effect of DI‐GM on PID risk was mediated by inflammatory dietary patterns, with higher DII scores indicating more pro‐inflammatory diets. Statistical analysis and data handling were conducted using R (version 4.4.0) and Zstats (version 3.0) software. Statistical significance was set at *p* < 0.05.

## 3 Results

### 3.1 Characteristics of Participants

As shown in Table 1, of the 4539 eligible participants, 255 women had PID. Those in the PID cohort were generally older, non‐Hispanic Black, and had a higher BMI, lower poverty income ratio (PIR), reduced educational attainment, decreased high‐density lipoprotein cholesterol (HDL‐C) levels, elevated fasting blood glucose levels, and a greater prevalence of conditions such as divorce, separation, menstrual irregularity, smoking, alcohol consumption, and hypertension than those in the non‐PID cohort. Moreover, the mean DI‐GM value was significantly lower in the PID group than in the non‐PID group (4.65 vs. 5.11, *p* < 0.001). Conversely, the DII level was significantly higher in the PID group than in the non‐PID group (1.70 vs. 1.07, *p* < 0.001). Furthermore, the prevalence of PID among participants significantly decreased from Q1 to Q4 (*p* = 0.013, Table S1), with notably lower rates in Q3 and Q4 (4.71% and 4.20%, respectively) than in Q1 and Q2 (7.76% and 6.57%, respectively). These observed variations suggest that the potential association between DI‐GM and PID requires further investigation.

**TABLE 1:** Basic characteristics of the participants according to PID status^a^.

Variables	Total (*n* = 4539)	PID (*n* = 2,55)	Non‐PID (*n* = 4284)	*p*
Age, mean (SE), year	39.25 (0.26)	42.70 (0.61)	39.06 (0.27)	**< 0.001**
BMI, mean (SE), kg/m^2^	29.78 (0.23)	31.80 (0.59)	29.66 (0.23)	**0.001**
PIR, mean (SE)	2.92 (0.05)	2.20 (0.13)	2.96 (0.05)	**< 0.001**
Triglyceride, Mean (SE), mg/dL	127.30 (2.02)	137.51 (6.17)	126.72 (2.08)	0.094
Fasting blood glucose, Mean (SE), mg/dL	102.27 (0.44)	105.70 (1.61)	102.07 (0.42)	**0.022**
HDL‐C, Mean (SE), mg/dL	58.18 (0.36)	54.03 (1.16)	58.42 (0.37)	**< 0.001**
DI‐GM, mean (SE)	5.08 (0.04)	4.65 (0.12)	5.11 (0.04)	**< 0.001**
DII, Mean (SE)	1.10 (0.06)	1.70 (0.13)	1.07 (0.06)	**< 0.001**
**Race, *n* (%)**				**0.033**
Mexican American	734 (9.82)	22 (5.38)	712 (10.07)	
Other Hispanic	489 (6.99)	24 (6.55)	465 (7.02)	
Non‐Hispanic White	1565 (61.14)	97 (57.00)	1468 (61.38)	
Non‐Hispanic Black	1043 (12.96)	81 (18.45)	962 (12.64)	
Other Race	708 (9.09)	31 (12.62)	677 (8.88)	
**Marital status, *n* (%)**				**< 0.001**
Married	2064 (51.68)	100 (40.54)	1964 (52.32)	
Widowed	85 (1.64)	8 (3.55)	77 (1.53)	
Divorced	480 (10.31)	48 (20.90)	432 (9.71)	
Separated	176 (3.06)	19 (8.68)	157 (2.74)	
Never married	1195 (22.86)	44 (13.89)	1151 (23.38)	
Living with partner	538 (10.44)	36 (12.44)	502 (10.33)	
**Education level, *n* (%)**				**0.013**
Below high school	757 (11.38)	47 (18.73)	710 (10.96)	
High school	1020 (21.59)	59 (24.72)	961 (21.41)	
College or higher	2762 (67.03)	149 (56.54)	2613 (67.63)	
**Smoking status, *n* (%)**				**< 0.001**
Yes	1375 (33.03)	144 (62.30)	1231 (31.35)	
No	3164 (66.97)	111 (37.70)	3053 (68.65)	
**Drinking status, *n* (%)**				**< 0.001**
Yes	371 (8.37)	45 (20.28)	326 (7.69)	
No	4168 (91.63)	210 (79.72)	3958 (92.31)	
**Regular menstruation, *n* (%)**				**< 0.001**
Yes	3166 (67.01)	144 (51.77)	3022 (67.89)	
No	1373 (32.99)	111 (48.23)	1262 (32.11)	
**Hypertension, *n* (%)**				**< 0.001**
Yes	1044 (21.15)	100 (36.56)	944 (20.27)	
No	3495 (78.85)	155 (63.44)	3340 (79.73)	
**Diabetes, *n* (%)**				0.584
Yes	345 (6.30)	25 (7.14)	320 (6.25)	
No	4194 (93.70)	230 (92.86)	3964 (93.75)	

*Note:* Significant *p*‐values are bolded.

Abbreviations: BMI, body mass index; DI‐GM, dietary index for gut microbiota; DII, dietary inflammatory index; HDL‐C, high‐density lipoprotein cholesterol; PIR, poverty impact ratio.

^a^ Percentage estimates are nationally representative using survey weights.

### 3.2 Association between DI‐GM and PID

The correlation between the DI‐GM score and PID risk is presented in Table 2. Logistic regression analysis identified a significant inverse relationship between DI‐GM scores and PID risk. When DI‐GM was treated as a continuous variable, the odds ratio (OR) for Model 1 (M1) was 0.85 (95% CI = 0.78–0.93, *p* < 0.001). After adjusting for demographic factors (M2) and in the fully adjusted model (M3), the OR values remained significant (M2: OR = 0.87, 95% CI = 0.79–0.95, *p* = 0.005; M3: OR = 0.87, 95% CI = 0.79–0.96, *p* = 0.012). Further analysis based on DI‐GM score groupings corroborated these findings. The results indicated that the highest group (Q4) was significantly associated with a reduced risk of PID compared to the lowest group (Q1) across all three models (M1: OR = 0.52, 95% CI = 0.35–0.78, *p* = 0.002, *p for* trend = 0.003; M2: OR = 0.54, 95% CI = 0.35–0.84, *p* = 0.01, *p* for trend = 0.014; and M3: OR = 0.55, 95% CI = 0.35–0.87, *p* = 0.017, *p* for trend = 0.018). Notably, the trend analyses across all models demonstrated statistical significance (*p* < 0.05), further supporting the strong association between higher DI‐GM scores and a decreased risk of PID development.

**TABLE 2:** Adjustment association analysis between DI‐GM and PID.

Variables	Model 1		Model 2		Model 3	
	OR (95% CI)	*p*	OR (95% CI)	*p*	OR (95% CI)	*p*
DI‐GM (Continuous)	0.85 (0.78–0.93)	< 0.001	0.87 (0.79–0.95)	0.005	0.87 (0.79–0.96)	0.012
**DI‐GM group**						
Q1 (0–3, *n* = 866)	1.00 (Reference)		1.00 (Reference)		1.00 (Reference)	
Q2 (4, *n* = 983)	0.83 (0.53–1.31)	0.437	0.76 (0.49–1.20)	0.198	0.76 (0.47–1.22)	0.167
Q3 (5, *n* = 1076)	0.59 (0.37–0.92)	0.026	0.60 (0.39–0.94)	0.032	0.58 (0.36–0.94)	0.036
Q4 (≥ 6, *n* = 1614)	0.52 (0.35–0.78)	0.002	0.54 (0.35–0.84)	0.010	0.55 (0.35–0.87)	0.017
*p* for trend	0.003		0.014		0.018	

*Note:* Model 1: no adjusted. Model 2: adjusted for age, ethnicity, education level, PIR, and marital status. Model 3: further adjusted for BMI, smoking, drinking, diabetes, hypertension, regular menstruation.

### 3.3 RCS And Threshold Analysis

**FIGURE 2:**
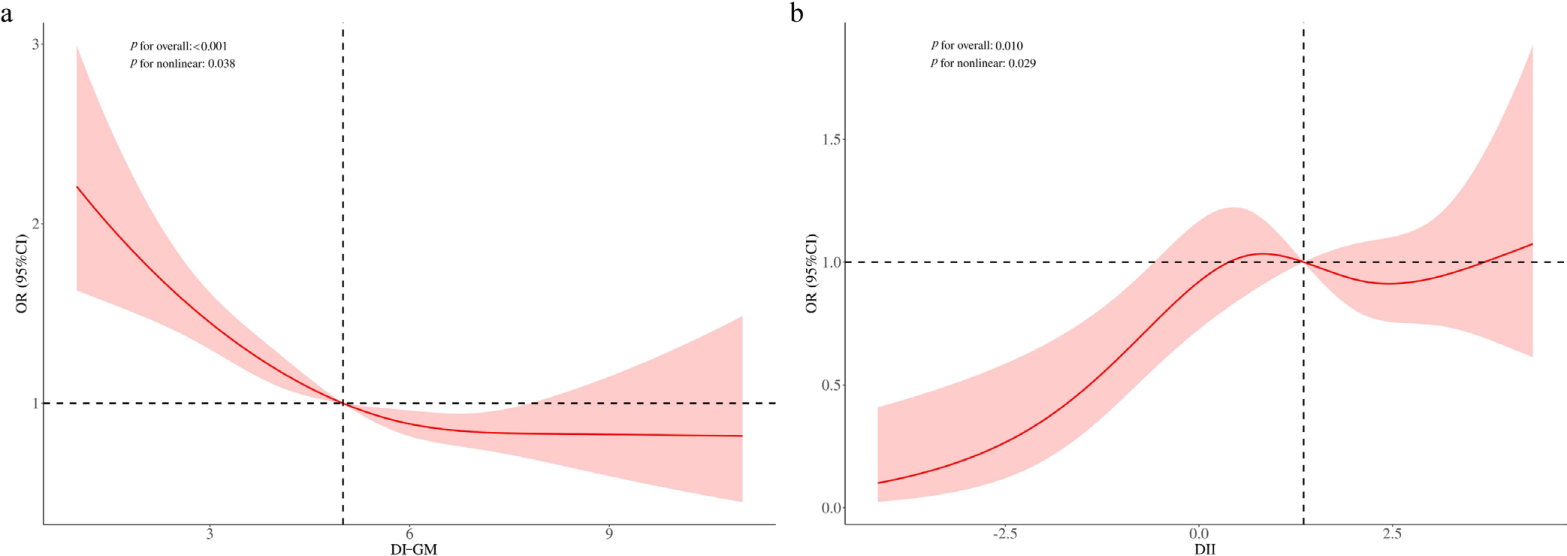
RCS analysis illustrating the association between DI‐GM scores and PID risk (a), as well as the relationship between DII and PID risk (b). Adjusted for age, ethnicity, education level, PIR, marital status, smoking, drinking, BMI, diabetes, hypertension, and regular menstrual cycle.

The RCS was used to illustrate the association between DI‐GM scores and PID risk. A nonlinear relationship was detected between DI‐GM and PID (Figure 2a; *p* for nonlinearity = 0.038). To further investigate this relationship, a weighted two‐segment linear regression model and recursive algorithm were applied to perform threshold effect analysis. This analysis identified an inflection point at a DI‐GM score of 4.3, with a log‐likelihood ratio test indicating significance at *p* < 0.001. These findings suggest that for DI‐GM scores from zero to 4.3, each unit increase in the DI‐GM score was associated with a 26% reduction in PID risk (OR = 0.74, 95% CI = 0.68–0.82, *p* < 0.001; Table 3).

**TABLE 3:** Threshold effect analysis of DI‐GM and PID^a^.

Outcome	Effect OR (95% CI)	*p*
**DI‐GM**		
Model 1	0.89 (0.86–0.93)	< 0.001
**Model 2**		
Inflection point	4.30	
< 4.30	0.74 (0.68–0.82)	< 0.001
≥ 4.30	0.99 (0.91–1.06)	0.720
*p* for likelihood test		< 0.001

*Note:* Model 1: fitting model using standard linear regression. Model 2: fitting model using two‐piecewise linear regression.

^a^ Adjusted for age, ethnicity, education level, PIR, marital status, smoking, drinking, BMI, diabetes, hypertension, regular menstruation.

### 3.4 Subgroup Analysis

**FIGURE 3:**
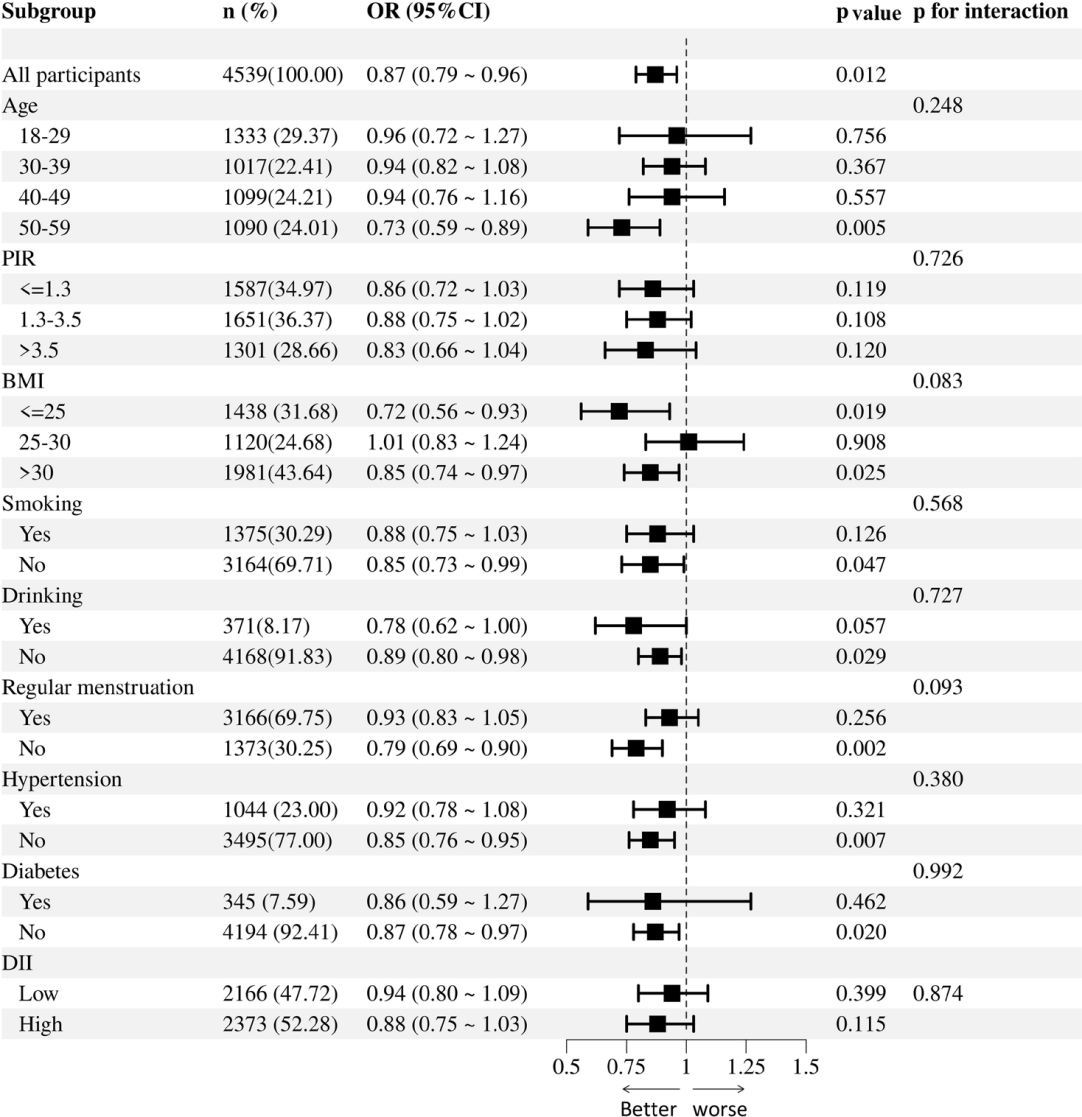
Forest plot of stratified analysis and interaction effects on the association between DI‐GM and PID. The model was adjusted for age, ethnicity, education level, PIR, marital status, smoking, drinking, BMI, diabetes, hypertension, and regular menstrual cycle.

To delve deeper into heterogeneity, stratified analyses were conducted, and the interaction *p*‐value was assessed. Figure 3 illustrates the stratified association between DI‐GM and PID risk. No significant interactions were observed between the covariates and DI‐GM for the PID risk. A significant inverse relationship between DI‐GM and PID risk was consistently observed across the various subgroups. These subgroups included individuals aged 50–59 years, BMI ≤ 25 or > 30, menstrual irregularities, and those without smoking, drinking, diabetes, and hypertension (all *p* < 0.05). These extensive subgroup analyses provide robust evidence of a consistent association between DI‐GM scores and PID risk across diverse populations.

### 3.5 Association Between DII, DI‐GM, and PID

The results of the multivariable weighted linear regression analysis between DI‐GM and DII, and the multivariable weighted logistic regression analysis between DII and PID are presented in Table 4. In the fully adjusted model, DI‐GM exhibited a negative correlation with the DII (β = −0.36, 95% CI = −0.41, −0.31, *p* < 0.001), whereas the DII demonstrated a positive correlation with PID (OR = 1.12, 95% CI = 1.02–1.23, *p* = 0.028). In addition, a nonlinear relationship was detected between the DII and PID risk (Figure 2b; *p* for nonlinearity = 0.029). These findings suggest that the DII, which reflects anti‐ and pro‐inflammatory dietary patterns, may be associated with the relationship between DI‐GM and PID.

**TABLE 4:** Adjustment association analysis between DI‐GM, DII, and PID.

Variables	Model 1	Model 2	Model 3
**Between DI‐GM and DII**			
β (95% CI)	−0.40 (−0.45, −0.35)	−0.37 (−0.41, −0.32)	−0.36 (−0.41, −0.31)
*p* value	< 0.001	< 0.001	< 0.001
**Between DII and PID**			
OR (95% CI) *p* value	1.23 (1.11, 1.36) < 0.001	1.14 (1.03, 1.25) 0.014	1.12 (1.02, 1.23) 0.028

*Note:* Model 1: no adjusted. Model 2: adjusted for age, ethnicity, education level, PIR, and marital status. Model 3: further adjusted for BMI, smoking, drinking, diabetes, hypertension, regular menstruation.

### 3.6 Mediation Analysis

**FIGURE 4:**
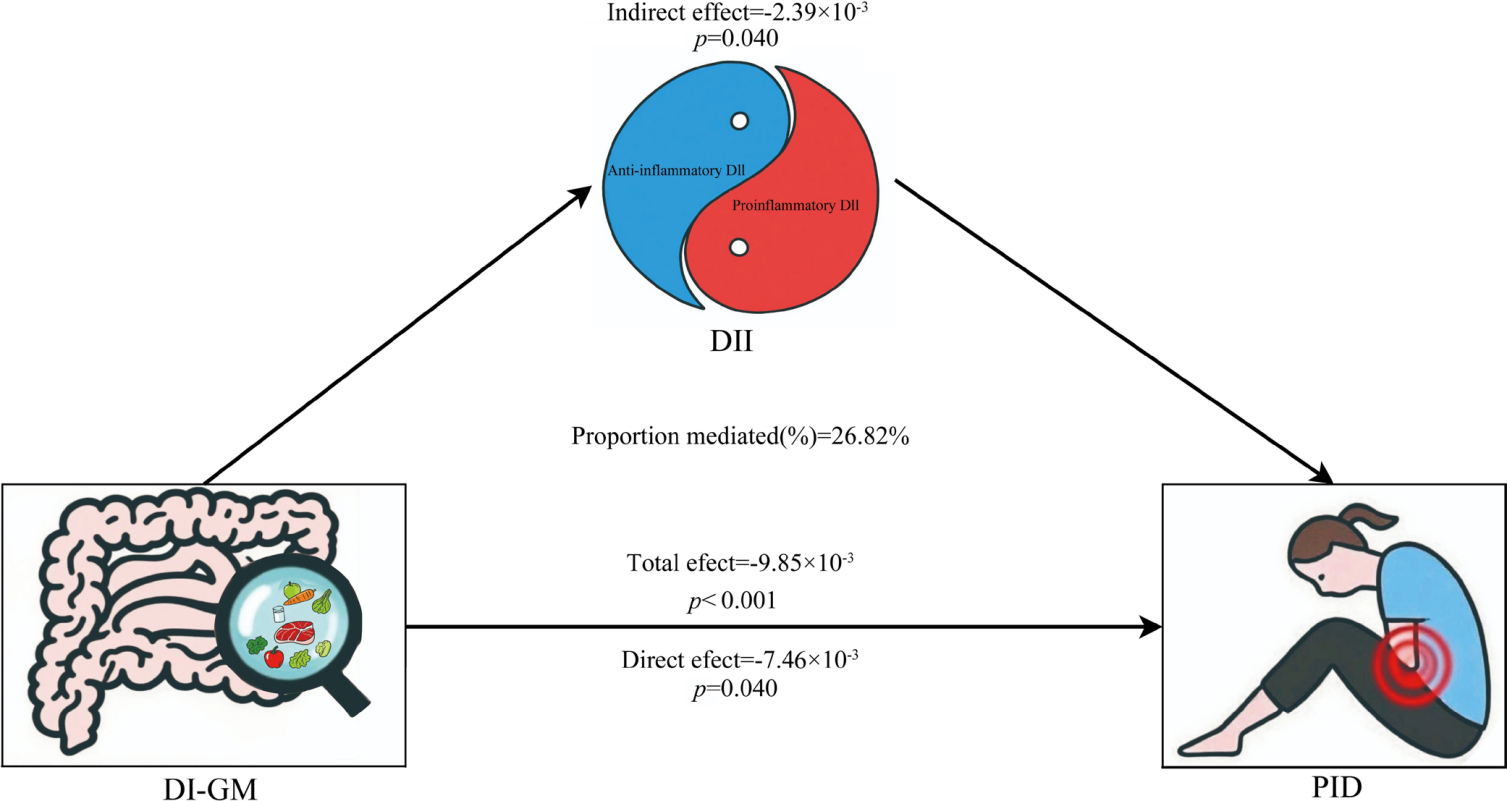
Analysis of the mediation of DII in the association between DI‐GM and PID.

To identify potential mediating variables in the relationship between DI‐GM and PID, we conducted a mediation analysis to examine the DII's role. The findings indicated that the mediating effect of inflammatory dietary patterns on the association between DI‐GM and PID in the fully adjusted model was −2.39 × 10^−3^ (*p* = 0.04), with a mediation proportion of 26.82% (Figure 4).

## 4 Discussion

In this study, we identified a significant correlation between DI‐GM and PID. Our findings showed that the non‐PID group had notably higher DI‐GM scores than the PID group. Higher DI‐GM scores were significantly inversely associated with the risk of developing PID. The healthy dietary pattern represented by a higher DI‐GM exerted a protective effect in subgroups aged 50–59 years, individuals with BMI ≤ 25 or > 30, menstrual irregularities, and those without smoking, drinking, diabetes, and hypertension. The RCS analysis revealed a nonlinear relationship between DI‐GM scores and PID risk, indicating a significantly reduced risk of PID when the DI‐GM score ranged from zero to the inflection point of 4.3. Furthermore, a negative correlation was identified between DI‐GM and DII, whereas a positive nonlinear relationship was observed between DII and PID risk. The mediation analysis results indicated that the mediation proportion of inflammatory dietary patterns accounted for 26.82% of the association between DI‐GM and PID.

Recent research indicates that adherence to healthy dietary patterns, as reflected by elevated DI‐GM scores, may optimize gut microbial community structure and enhance intestinal barrier function (Kase et al. 2024). The DI‐GM is more adaptable than traditional dietary indices and is more suitable for personalized dietary recommendations (Kirkpatrick et al. 2018; Liu and Huang 2025; Si et al. 2025; Zhang, Yang, et al. 2025). Higher DI‐GM scores are generally linked to diets abundant in dietary fiber and antioxidants, which facilitate the proliferation of beneficial bacteria such as *Bifidobacterium* and *Lactobacillus* (Bolte et al. 2021; Shen et al. 2025; Valdes et al. 2018). These beneficial bacteria not only mitigate pathogenic colonization through competitive inhibition but also secrete short‐chain fatty acids (SCFAs), including acetate, butyrate, and propionate. These SCFAs enhance the integrity of tight junctions between intestinal epithelial cells and reduce intestinal permeability (Thulasinathan et al. 2025). Mechanically, enhancing the function of the intestinal barrier is an effective strategy to prevent the translocation of opportunistic gut pathogens, thereby inhibiting their migration to the pelvic cavity through blood or lymphatic circulation and consequently reducing the risk of PID (Dong et al. 2025; Ji et al. 2024; Yin et al. 2025). Additionally, studies have established that foods can affect the structure and function of gut microbiota through defined mechanisms. β‐glucan in whole grains is metabolized into SCFAs, promoting SCFA‐producing bacteria like *Clostridium spp*. (Bach Knudsen 2015; Kim 2021). Chlorogenic acid in coffee inhibits pathogenic bacterial adhesion while enhancing microbiota α‐diversity (Rojas‐Gonzalez et al. 2022). Nitrite in processed meats converts to carcinogenic nitrosamines and reduces the abundance of *Lactobacillus* (Zhang et al. 2023). Fructose in sugar‐sweetened beverages disrupts microbiota balance, increasing pro‐inflammatory bacteria such as *Proteobacteria* (Shon et al. 2023).

Subgroup analyses indicated that the negative association persisted consistently across most demographic and health‐related categories, with no statistically significant interactions between DI‐GM and covariates. Hormonal differences may significantly influence the interactions between DI‐GM and adult females, affecting the risk of PID (Vemuri et al. 2019). Additionally, individuals with obesity, smoking, alcohol consumption, and conditions such as diabetes and hypertension are predisposed to a heightened risk of chronic diseases, including PID (Herup‐Wheeler et al. 2024; M. Li and McDermott 2015; Ng et al. 2020; Okoth et al. 2023; D. Wang and Xiong 2025). This increased risk is attributed to the modulatory effects of aging, inflammation, protein intake, and metabolic disorders (Dodd and Menon 2022; Jang et al. 2024; Rizzetto et al. 2018). The observed correlation between elevated DI‐GM scores and a reduced risk of PID underscores the potential efficacy of dietary interventions in enhancing the gastrointestinal health of older adult women. Notably, the analysis of the DI‐GM score threshold effect revealed a more pronounced protective association when DI‐GM ≥ 0 and < 4.3. Specifically, each unit increase in the DI‐GM score corresponded to a statistically significant 26% reduction in PID risk. The DI‐GM calculation method suggests that a higher score correlates with a decrease in the consumption of dietary components that are high in energy density, such as red meat and high‐fat milk (Kase et al. 2024). Our findings indicate that sufficient protein consumption significantly mitigates the risk of developing PID. These results imply that the optimal approach for older adults involves balancing protein intake with beneficial components for gut health, as indicated by the DI‐GM, while minimizing the unfavorable components. This balance is essential for the immune system, as it is regulated by energy and nutritional intake.

The DII was developed to quantitatively assess the impact of dietary patterns on health. It encompasses forty‐five pro‐ and anti‐inflammatory food parameters and six inflammatory biomarkers (Shivappa et al. 2014). The mediation analysis results indicated that the inflammatory dietary patterns significantly mediated the association between DI‐GM and PID, accounting for 26.82% of the mediation proportion. These findings align with those of previous studies, suggesting that a higher DII is associated with an increased risk of PID (Ma et al. 2024). Interestingly, recent studies have shown that anti‐ and pro‐inflammatory dietary patterns mediate the association between DI‐GM, sleep disorders, and sarcopenia (Gong et al. 2025; Y. Li et al. 2025). These findings suggest that restoring gut microbiota composition and its regulatory metabolites through dietary interventions may help prevent or ameliorate PID.

Additionally, it is imperative to consider the potential reverse causation. Women diagnosed with PID may modify their dietary practices as a consequence of the disease or antibiotic treatment. PID is frequently associated with gastrointestinal symptoms such as nausea and vomiting, which may lead patients to opt for light, easily digestible foods, reduce their overall food intake, or alter the frequency of their meals (Brunham et al. 2015). Moreover, when patients with PID undergo antibiotic treatment, the antibiotics can disrupt the equilibrium of normal intestinal flora, resulting in diminished digestive and absorptive functions and a compromised intestinal barrier (Savaris et al. 2020). Consequently, patients may adjust their diets to mitigate intestinal discomfort, thereby altering their original dietary habits.

This study has several limitations that should be considered. First, its cross‐sectional design does not allow for the establishment of a causal relationship between DI‐GM and PID, highlighting the need for future prospective cohort studies or randomized controlled trials to confirm this association. Second, dietary patterns assessed using questionnaires may introduce recall bias, potentially affecting the accuracy of the DI‐GM scores. Third, given that a significant portion of PID is likely subclinical, there is a risk of selection bias in the inclusion of PID cases in the analysis. Fourth, the diagnosis of PID in the questionnaire survey lacked definitive clinical validation and failed to distinguish between acute and previous PID. Finally, this study did not investigate the specific roles of certain gut microbial species or metabolites, necessitating follow‐up research using metagenomics and metabolomics to thoroughly analyze the key microbial and metabolic mechanisms underlying PID pathogenesis.

Despite these limitations, the findings of this study are significant. The marked association between DI‐GM and PID suggests that optimizing gut microbiota through dietary modification may be a potential strategy for preventing PID. Future research should focus on developing personalized dietary intervention programs for high‐risk populations and enhancing public health education to improve awareness of diet‐microbiota‐health relationships, offering new dietary approaches for PID prevention and control. Additionally, as a modifiable dietary indicator, DI‐GM has greater intervention feasibility than non‐modifiable PID risk factors, suggesting that it may play a key role in the primary prevention of PID.

## 5 Conclusion

The present study revealed a notable negative correlation between DI‐GM and PID risk in females. Notably, the connection between the DI‐GM scores and PID exhibited nonlinear trends. As an innovative dietary quality index that indicates gut microbiota diversity, additional studies and interventions utilizing DI‐GM could aid in developing strategies to prevent and reduce the risk of PID.

## Author Contributions

Yanjing Bao and Xianyue Hu contributed to data analysis and interpretation and were responsible for drafting the manuscript. Wenfeng Hua and Tianyang Gao were involved in the conception and design of the study and provided editorial revisions to the manuscript. All the authors have reviewed and approved the final version of the manuscript. No other individuals contributed substantially to this study.

## Funding

This work was supported by Science and Technology Projects of Guangzhou, 2024A03J1059, 2024A03J0990.

## Ethics Statement

The NHANES protocols were approved by the National Center for NCHS Ethics Review Board. This study adhered to local legislation and institutional requirements. All participants provided written informed consent to participate in the study.

## Conflicts of Interest

The authors declare no conflicts of interest.

## Supporting information


**Data S1:** fsn371563‐sup‐0001‐TableS1.docx.

## Data Availability

The original data presented in the study are openly available at the National Health and Nutrition Examination Survey at https://wwwn.cdc.gov/nchs/nhanes/.
