# Supplementary material for: Association Between Dietary Index for Gut Microbiota and the Risk of Pelvic Inflammatory Disease: Mediating Role of Anti‐ and Pro‐Inflammatory Dietary Patterns
Source: Food Sci Nutr. 2026 Feb 19;14(2):e71563. doi: 10.1002/fsn3.71563 (PMC12920683; doi:10.1002/fsn3.71563)
Supplement: Supplementary file 1 — Data S1: fsn371563‐sup‐0001‐TableS1.docx. [file FSN3-14-e71563-s001.docx]

| **Table S1. Basic characteristics of the study population based on** **DI-GM scores*.** | | | | | |
| --- | --- | --- | --- | --- | --- |
| **Variable** | **Q1**  **(0-3, n=866)** | **Q2**  **(4, n=983)** | **Q3**  **(5, n=1076)** | **Q4**  **(≥6, n=1614)** | ***p* value** |
|  |  |  |  |  |  |
| **Age, mean (SE), year** | 39.25 (0.26) | 37.64 (0.45) | 39.01 (0.62) | 38.64 (0.51) | <0.001 |
| **BMI, mean (SE), kg/m^2^** | 29.78 (0.23) | 31.10 (0.43) | 30.66 (0.34) | 29.67 (0.42) | <0.001 |
| **PIR, mean (SE)** | 2.92 (0.05) | 2.63 (0.09) | 2.51 (0.09) | 2.92 (0.07) | <0.001 |
| **Triglyceride,**  **mean (SE), mg/dL** | 127.30 (2.02) | 121.35 (3.29) | 135.82 (3.35) | 134.00 (5.85) | 0.002 |
| **Fasting blood glucose, mean (SE), mg/dL** | 102.27 (0.44) | 102.56 (0.84) | 105.32 (1.27) | 101.95 (0.82) | 0.025 |
| **HDL-C,**  **mean (SE), mg/dL** | 58.18 (0.36) | 55.85 (0.63) | 55.87 (0.63) | 58.48 (0.66) | <0.001 |
| **DII, Mean (SE)** | 1.88 (0.05) | 1.70 (0.08) | 1.16 (0.10) | 0.40 (0.07) | <0.001 |
| **Race, n (%)** |  |  |  |  | <0.001 |
| Mexican American | 128 (10.19) | 162 (10.44) | 188 (10.35) | 256 (9.00) |  |
| Other Hispanic | 98 (8.08) | 117 (8.30) | 103 (6.16) | 171 (6.35) |  |
| Non-Hispanic White | 266 (55.65) | 329 (58.10) | 386 (61.88) | 584 (64.72) |  |
| Non-Hispanic Black | 266 (19.17) | 256 (15.65) | 230 (11.80) | 291 (9.50) |  |
| Other Race - Including  Multi-Racial | 108 (6.91) | 119 (7.50) | 169 (9.82) | 312 (10.43) |  |
| **Marital status, n (%)** |  |  |  |  | 0.015 |
| Married | 342 (45.45) | 404 (48.95) | 508 (54.96) | 811 (53.82) |  |
| Widowed | 19 (1.90) | 23 (1.93) | 17 (1.16) | 26 (1.68) |  |
| Divorced | 86 (10.98) | 116 (12.08) | 109 (8.06) | 169 (10.50) |  |
| Separated | 26 (2.63) | 42 (3.07) | 39 (2.32) | 68 (3.72) |  |
| Never married | 268 (25.33) | 275 (24.29) | 280 (23.01) | 373 (20.93) |  |
| Living with partner | 125 (13.70) | 123 (9.67) | 123 (10.48) | 167 (9.36) |  |
| **Education level, n (%)** |  |  |  |  | <0.001 |
| Below high school | 148 (12.61) | 198 (14.36) | 199 (12.81) | 212 (8.37) |  |
| High school | 231 (27.19) | 244 (27.68) | 257 (22.89) | 288 (15.09) |  |
| College or higher | 487 (60.20) | 541 (57.96) | 620 (64.29) | 1114 (76.54) |  |
| **Smoking status, n (%)** |  |  |  |  | 0.045 |
| Yes | 264 (31.95) | 310 (35.18) | 353 (36.35) | 448 (30.30) |  |
| No | 602 (68.05) | 673 (64.82) | 723 (63.65) | 1166 (69.70) |  |
| **Drinking status, n (%)** |  |  |  |  | 0.485 |
| Yes | 73 (7.28) | 68 (7.76) | 93 (9.37) | 137 (8.55) |  |
| No | 793 (92.72) | 915 (92.24) | 983 (90.63) | 1477 (91.45) |  |
| **Regular menstruation,**  **n (%)** |  |  |  |  | 0.110 |
| Yes | 618 (69.67) | 661 (63.30) | 771 (69.61) | 1116 (66.15) |  |
| No | 248 (30.33) | 322 (36.70) | 305 (30.39) | 498 (33.85) |  |
| **Hypertension, n (%)** |  |  |  |  | 0.069 |
| Yes | 211 (22.06) | 240 (25.50) | 253 (19.47) | 340 (19.53) |  |
| No | 655 (77.94) | 743 (74.50) | 823 (80.53) | 1274 (80.47) |  |
| **Diabetes, n (%)** |  |  |  |  | 0.006 |
| Yes | 74 (8.07) | 83 (8.26) | 87 (6.65) | 101 (4.27) |  |
| No | 792 (91.93) | 900 (91.74) | 989 (93.35) | 1513 (95.73) |  |
| **PID, n (%)** |  |  |  |  | 0.013 |
| Yes | 69 (7.76) | 56 (6.57) | 52 (4.71) | 78 (4.20) |  |
| No | 797 (92.24) | 927 (93.43) | 1024 (95.29) | 1536 (95.80) |  |
| BMI, body mass index; PIR, poverty impact ratio; HDL-C, high-density lipoprotein cholesterol; DI-GM, dietary index for gut microbiota; DII, dietary inflammatory index.  ^*^Percentage estimates are nationally representative using survey weights. | | | | | |
